# Pott’s Disease as Illustrating the Principles of Diagnosis

**Published:** 1884-04

**Authors:** C. E. Webster


### ARTICLE II.

### POTT'S DISEASE AS ILLUSTRATING THE PRINCIPLES OF DIAGNOSIS. By C. E. WEBSTER, M.D.

Perhaps I more than others can appreciate the importance of early diagnosis in the disease under consideration, and as I walk the streets, I am well assured by many living proofs that in the past there has been negligence in this regard.

The busy general practitioner, with his mind harassed by a thousand cares, is liable to overlook the critical condition of some fretful, sickly child until the golden opportunity is lost, and he, the child, is burdened with the perpetual curse of an ineradicable deformity.

In order to present a paper of sufficient breadth of scope to be of general interest, I have incorporated the notes on Pott's Disease, which are for the most part compiled from standard authors, in an essay upon the subject of diagnosis. If this paper succeeds in awakening sluggish memories, and thus some *one* is saved by early treatment from lifelong misfortune, it will have well fulfilled its purpose.

The treatment of a case without a diagnosis is like waging war without a fortified base of operations. It can be done, and successfully, too, but it is not the highest refinement of art by any means. The most skilful physician, like the best trained general, may be forced into action without time for intrenchment, and if the odds are not too great against him, he may win the day. Uneducated doctors, like untaught savages, kill, take captive, and are victorious. The one protects his nakedness with a flimsy shield, and the other his ignorance with a fanciful diagnosis. But neither the success in emergency of the expert, or the prosperity of the quack, can justify departure in ordinary cases from the common course of procedure. Therefore it may be assumed that the recognition of the disease treated is the first requsite to successful practice.

Each disease has an individuality of its own, which is preserved through all its varieties and amid all its complexity of symptoms. Were this not the fact, scientific medicine would be impossible, for classification is the first necessity of exact science, and the identification of species is the first requisite to classification. Among diseases, species may be ill defined, may run into each other by innumerable varieties, or a particular case may present some startling anomaly; but strongly marked individual differences are not inconsistent with a specific resemblance, and the most hazy nebula has as real an entity as the most sharply defined planet.

Admitting the entity of a given disease, the question arises, in what does that entity consist? It is like the individuality of a face. Some faces have a characteristic feature; a nose, an eyebrow, a chin that is characteristic, and that face is remembered by that feature. So certain diseases have pathognomonic symptoms; as the periodicity of the attack in intermittent fever, and such diseases can be diagnosticated by their pathognomonic symptoms. In general, faces are remembered not by a process of analysis; not by their individual features, but as single units of cognition. You may know a person intimately, but cannot tell the exact shade of his hair or eyes, the shape of his nose, or the cut of his beard. Let him shave; let time furrow his face with wrinkles and pull out half his fading hair, you will still recognize him, though not a feature but has suffered some appreciable change.

It is the same with most diseases. It is often said of such and such a doctor, "he has a peculiar knack of diagnosis." In what does his peculiar excellence lie? He is old and little educated in the schools; his sight is dim, his hearing dull; he cannot appreciate fine points of auscultation and percussion. The Faradaic current is beyond his ken. He can hardly assign a reasonable cause for a single symptom, but he unconsciously coördinates those symptoms that he observes into a single unit of consciousness, giving each feature its due prominence in the picture drawn, and by taking a telescopic view of the case, and by comparing this with similar pictures of other cases that he has seen, he gives his diagnosis. His view is broad. He neglects much detail, but he sees the case as a whole. In general practice, it is this telescopic power of the mind that makes a diagnostician. There is more to diagnosis than this, however.

In common life, recognition of an individual is sufficient. In a court of law, identification becomes necessary. In ordinary practice, a snap diagnosis may be sufficient. In the scientific study of disease, in commencing treatment in a fatal or chronic malady, or in a formal consultation, an exact diagnosis is necessary. This may be reached by one of two methods—either directly, by comparing the symptoms of the case with the described symptoms of the disease, or indirectly by the differential method, considering all possible maladies that might exist in the case, and eliminating them singly by the direct method, giving the preference to the residual disease.

To illustrate these principles, let us consider the early recognition of Pott's Disease.

A glance at the patient may be sufficient for the provisional or snap diagnosis. You see a sickly child with an anxious expression of face, moving cautiously, seeking support from external objects, and carrying its trunk erect. Throw some object on the floor for the child to pick up. It stoops by flexing the legs, but still carries the body erect and steady, as though the spine was made of glass, and it was afraid of breaking it. The seeking of support, and carefulness respecting jar or strain of spine, are the most distinctive features, and may be variously modified according to the age of the patient, the location and acuteness of the nflammation, and other circumstances of the case.

With the first glance you have recognized spinal caries, but in so serious a matter an opinion should not be given until the exact diagnosis is reached, which necessitates a history of the illness and a thorough examination. If unwilling to assume the responsibility, or unable from press of business to take the trouble, send the case to one who will, for you have before you for treatment a patient destined to live in spite of the grossest negligence, but whose future happiness and usefulness alike depend upon the care and fidelity of some one who for years shall enforce those simple rules of life, and adapt with care and accuracy the necessary appliances.

I can well assure you, gentlemen, that highway robbery were a much more honorable mode of obtaining wealth, than by careless diagnosis, procrastinating measures, or insufficient treatment, to rob a child of the slight chance of reaching that normal physical development, which is a prime requisite for maintaining his physical, mental and moral symmetry.

The history of the case will vary according to the variety of the disease. It is not intended to give a classification of these varieties, therefore it will suffice to mention all the symptoms likely to occur, premising that the absence of symptoms, or the inversion of their order, does not preclude the possibility of spinal caries.

A prodromal period, or period of incubation, is to be expected. During this stage, which may date from some sickness or injury, or may arise without any assignable cause, and be of several months' duration, the patient shows an indifference to his ordinary amusements and an unnatural fretfulness. He may become awkward in his movements, stumbling in his gait, or assume unlikely attitudes. There may be constitutional symptoms, as loss of appetite, anæmia, and a rise of temperature. There may be nervous symptoms suggestive of paralysis, as weakness, numbness, or tingling sensations in the lower extremities, with irritability of the bladder. If the disease is high in the column, gastric irritation, with a sensation of constriction of the chest. If upper cervical, difficult deglutition, choreaic movements of muscles of the neck, numbness of the arms, and laryngeal cough, or a choking sensation.

Pain is one of the most unreliable of all the symptoms. It is generally located anywhere, rather than at the seat of the disease, and many patients are entirely free from it. It is thought to be caused by the irritation of the spinal nerves at the point of their exit from the foramina, and is referred to the ultimate termination of those nerves. When it occurs it may be constant, if local; intermittent, if remote from the spine; and be increased by fatigue or sudden jar. Acute pain is suggestive of a suppurative inflammation, while a dull ache is more characteristic of dry caries. The location of the pain indicates the location of the disease. If thoracic, the disease is cervical. If in the abdomen and lower part of thorax, the disease is thoracic. While if through the thighs, the disease is lumbar.

A satisfactory history of the case having been taken, the patient is to be stripped and inspected. Flexion of the spine is avoided, and a constant effort made by the patient to support himself. He leans upon the furniture, rests his hands on his thighs, or lies across some object. His movements are constrained in order to avoid jar. He shuffles in his gait. From reflex muscular spasm there may be a partial flexure of the thighs, and a local stiffening in the portion of the spine affected. Torticollis may occur. The head may be thrown back, and the shoulders raised. The respiration may be grunting in character, with limited motion of some of the ribs. The first evidence of the location of the disease visible to the eye may be a slight flattening of the normal curves of the spine, with stiffening of the affected part, an indication of little value in young children when the curves are not developed. There may be a slight bulging, or a slight lateral angle in the line of the spinous processes at the diseased point, and finally, as the commencement of the deformity, there is the formation of a knuckle produced by the tilting up of the spine of the vertebra whose body is most eroded.

Follow the inspection by a physical examination to establish the diagnosis and locate the disease. Let the patient jump down from a slight elevation, as a low stool. This concussion may produce a cry of pain, a slight confusion of ideas, the child looking surprised, or even a fall. Another method of producing the same result is to press down on the head and shoulders, producing a cry and muscular spasm. These are both rather dangerous experiments. Rubbing the back briskly with the knuckles makes the vetebral spines stand out in red spots. This may assist in detecting any displacement. Pain may be produced at the point of disease by percussion, firm pressure, compression of the chest, crowding the heads of the ribs against the diseased vertebra, a piece of ice or a thimblefnl of hot water passed down the back; and lastly, by lateral flexion of the spine, the patient lying on his face, and the body being grasped by the pelvis and shoulders. If, with the patient standing erect, the palm of the hand be placed against the suspected region, and then the spine bent in various directions, any local stiffening can be detected, the neighboring healthy parts being felt to move. The heat of the inflammation can sometimes be detected by the hand or by the surface thermometer. There may also slight diffuse swelling. If the patient has pain of any sort, impeded respiration, or any uncomfortable sensation, lay him across the knees, on a table, or place him in a spiral swing and make firm gentle extension or partial suspension. Observe if his symptoms are relieved. If he gives a sigh of relief and breathes freely while there is a cessation of his pain, and if the reverse of these maneuvers, by pressing the diseased bones more closely together, aggravates his symptoms, the diagnosis may be considered as fully established, and a plan of treatment adopted.

There are cases where the first symptom noticed is the knuckle of the commencing deformity, and others where paraplegia or abscess may occur without deformity. In obscure cases where the direct diagnosis is difficult, or in any case where especial accuracy is desired, the following table for differental diagnosis will be of service. Error is case of some of the diseases mentioned is much too common, and an occasional reference to such a table, though it be far from exhaustive, will be a valuable aid to the memory :

Sacro-Iliac Disease.—Resembles hip disease.

Torticollis.—If from cervical caries the patient supports the head with hand. Palpation detects thickening and tenderness of vertebra.

Superficial Caries.—Constitutional or traumatic causes, abscess, no deformity.

Aneurism of Aorta.—Deformity slight, not angular, signs of aneurism.

(Other Tumors.—Either inter-thoracie or abdominal; deformity not angular; physical diagnosis of tumor.—Dr. E. W. Andrews).

Disease of Kidney.—Differentiate by the urine.

Lateral Curvature.—Deformity not angular; no stiffness.

Rachitis.—Deformity not angular; affects other portions than the spine.

Muscular Weakness.—Deformity obliterated by lying down.

Hernia.—Sudden disappearance of tumor, with absence of spinal symptoms.

Paraplegia.—Without deformity may be ascribed to Pott's Disease if there are spinal symptoms and no other assignable cause.

Diseases of Childhood.—With a long prodromal stage are to be differentiated by the concomitant symptoms. (Acute, with local pain, to be differentiated by the concomitant symptoms and the results of treatment.—Dr. Graham).

Authorities.—Bauer, Brodie, Paget, Sager, Shaffer, Shaw, Taylor, and others.

Theories of treatment and facts of general science can be stored in text-books; they may be held in memory, or, when needed, sought by reference, as is most convenient; but it is the duty of the student, ere he becomes a practitioner, to become at least tolerably expert in the snap diagnosis of common diseases, if not invincible in exact diagnosis.

How are these powers to be acquired? By only one process—by the study of disease; by noting the gross appearance of disease as well as its most minute features; by clinical practice, supplemented by careful reading, and by acquiring the greatest possible proficiency in all those methods of physical exploration, without which scientific medicine would be an impossibility. Many a young graduate enters the sick-room of his first patient with trembling and abject fear. He has learned to auscultate and percuss with an instructor at his elbow, and to listen by the bedside in the hospital to an exact description of a patient's symptoms, but to interrogate and examine a patient with a view to identifying his disease and relieving his sufferings, is a thing to him as strange as were mounted horsemen to the American aborigines.

At the commencement of one's course, if he could learn the practical duties of a nurse and accustom himself to attendance on the sick, he would have done far better preliminary work than if he had mastered every department of natural science, for then the clinic would be real practice, the didactic lecture an illumination of intelligible pictures and not a weird phantasmagoria of the unreal. While the work of pathology would become the survey of old battle fields, the study of the historic part which is to fit him for coming victories,

In conclusion, let me quote my honored teacher in anatomy, Dr. Oliver Wendell Holmes, who, from the eminence attained by a life of philosophical labor, sends down this clue to guide the student groping among the underbrush of routine study: "I would not undervalue the branch I teach. I recognize the incidental importance of all the subsidiary branches which form a part of the curriculum of this and other schools. Do full justice to these, or you will not, probably, do justice to your more immediately practical studies. But your hardest study must be at the bedside. To go hastily from the library of old books and the laboratory of new experiments to the bedside of disease, is imitating the presumption of those rash profligates who, as Thomas Barton says, think they can take a 'leap out of Delilah's lap into Abraham's bosom.'"

